# Supplementary material for: The Role of Culture and Religion on Sexual and Reproductive Health Indicators and Help-Seeking Attitudes amongst 1.5 Generation Migrants in Australia: A Quantitative Pilot Study
Source: Int J Environ Res Public Health. 2021 Feb 2;18(3):1341. doi: 10.3390/ijerph18031341 (PMC7908584; doi:10.3390/ijerph18031341)
Supplement: Supplementary file 1 [file ijerph-18-01341-s001.pdf]

The Role of Culture and Religion on Sexual and Reproductive Health Indicators and Help-Seeking Attitudes Amongst 1.5 Generation Migrants in Australia: A Quantitative Pilot Study

Supplementary material

Text S1. Participant Information Sheet, Demographic Questions and Quantitative Survey

Culture Clash? An Investigation of the Constructions, Understandings and Experiences of Sexual and Rproductive Health from the Perspective of 1.5 Generation Migrants in Australia

Participant Information

Project Title: Culture Clash? An Investigation of the Constructions, Understandings and Experiences of Sexual and Reproductive Health from the Perspective of 1.5 Generation Migrants in Australia

Project Summary: This project aims to investigate the role of culture in constructions of sexual and reproductive health from the perspective of people who left their country of origin and migrated to Australia before completing puberty/adolescence. It also seeks to define the key aspects of one's culture and its messages about sexuality which help to shape how people within this cohort understand and experience sexual and reproduction health. In doing so the project seeks to make recommendations about the development and delivery of culturally appropriate sexual and reproduction health services towards increasing help-seeking and overall population health.

You are invited to participate in a research study being conducted by Dr Tinashe Dune, Lecturer in Interprofessional Health Science and Academic Course Advisor, Postgraduate Health Science, School of Science and Health - under the Supervision of Professor Janette Perz, Director for the Centre for Health Research, School of Medicine.

How is the study being paid for?
The study is being sponsored by the School of Science and Health Early Career Research Grant.

What will I be asked to do?
This research will involve two phases:
Online activities: First, you will be asked to complete a short questionnaire on your experiences with sexual and reproductive health and health services as well as any barriers or facilitators to access and utilisation of these services. You will then be asked to sort a number of statements related to sexual and reproduction health according to how much you agree or disagree with the statements. You can begin the study by following the link below. Your consent to participate is the online portion can be given after reading the online Participant Information and clicking on the 'Continue' button on that page.

Face-to-face interview: If you choose to participate the next phase will be a face-to-face interview following completion of the online sorting activity. This will be scheduled at a time which is convenient for you at one of the University of Western Sydney campuses. In the interview you will be asked to discuss and/or explain how you sorted the statements in the online activity thus allowing for a comprehensive and contextualised interpretation of your perspective. Consenting to participate in the first phase of this project does not mean you have consented to participate in the second phase.

If you would also like to participate in the face-to-face portion of this study, you be asked to provide your full name, phone number and email address at the end of the online activities. At the face-to-face interview you will be asked to sign the Participant Consent Form or orally indicate your consent to participate.

How much of my time will I need to give?

The online questions will take about 30 minutes to complete and the sorting activity will take approximately 30 minutes as well. The face-to-face interview will take approximately 1 hour.

What specific benefits will I receive for participating?
For your participation in both components of the research you will receive $25 cash upon the completion of the interview. Also participating in the study will provide you with the opportunity to examine and discuss issues relating to your understanding and experience of sexual and reproductive health from your perspective as a 1.5 generation migrant.

Will the study involve any discomfort for me? If so, what will you do to rectify it?
Participation is unlikely to involve any discomfort for you. However, if you do start to feel uncomfortable at any stage of the study, you will be free to take a break or terminate the online activity and/or interview at any time without repercussion.

How do you intend to publish the results?
Please be assured that only the researchers will have access to the raw data you provide.

The findings of the research will be published in peer-reviewed journals and presented at seminars and conferences.

*Please note that the minimum retention period for data collection is five years.

Can I withdraw from the study?
Participation is entirely voluntary and you are not obliged to be involved. If you do participate, you can withdraw at any time without giving a reason.

If you do choose to withdraw, any information that you have supplied will be deleted and/or destroyed.

Can I tell other people about the study?
Yes, you can tell other people about the study by providing them with the chief investigator's contact details. They can contact the chief investigator to discuss their participation in the research project and obtain an information sheet.

Data storage
There are a number of government initiatives in place to centrally store research data and to make it available for further research. For more information, see **http://www.ands.org.au/** and **http://www.rdsi.uq.edu.au/about**. Regardless of whether the information you supply or about you is stored centrally or not, it will be stored securely and it will be de-identified before it is made to available to any other researcher.

What if I require further information?
Please contact Dr Tinashe Dune should you wish to discuss the research further before deciding whether or not to participate.

Email: t.dune@uws.edu.au
Phone: 02 4620 3655

What if I have a complaint?
This study has been approved by the University of Western Sydney Human Research Ethics Committee. The Approval number is [enter approval number once the project has been approved]

If you have any complaints or reservations about the ethical conduct of this research, you may contact the Ethics Committee through the Office of Research Services on Tel +61 2 4736 0229 Fax +61 2 4736 0013 or email humanethics@uws.edu.au.

Any issues you raise will be treated in confidence and investigated fully, and you will be informed of the outcome.

If you agree to participate in this study please click on the 'Continue' button below. Doing so indicates your consent.

Continue

Demographic questions

1. What is today`s date? (dd/mm/yyyy)

   _ _/_ _/_ _ _ _

2. How do you identify:
   - o Male
   - o Female
   - o Other ______________________
3. What is your marital status:
   a. Single
   b. De facto
   c. Married
   d. Divorced
   e. Other ________________
4. Do you have children?
   a. Yes
      - ❑ How many? ______
      - ❑ How old are they? _______

   b. No
5. Where were you born?

   ____

6. How old are you?

   Please write your age in years: __________________

7. What is the language you speak primarily (first language)?
   - o English
   - o Not English, please specify___________________________
8. What year did you move to Australia? __________________
9. How old were you? __________________
10. Who did you come with? (Choose all that apply)
    - o Mother
    - o Father
    - o Siblings
    - o Grandparents

- Uncles and/or Aunts
- Family friend
- Extended family
- Other

11. What is your religious affiliation:
    a. No religion
    b. Christian
        - ❑ Which denomination? (please specify)____________________
    c. Catholic
    d. Buddhist
    e. Greek Orthodox
    f. Islamic
    g. Other (please specify) ____________________

Please indicate how you perceive the following:

12. I identify most with the culture and values from my country of origin
    - Strongly agree
    - Mostly agree
    - Neutral
    - Mostly disagree
    - Strongly disagree
13. I identify most with Australian culture
    - Strongly agree
    - Mostly agree
    - Neutral
    - Mostly disagree
    - Strongly disagree
14. I have strong relationships with my community based on my culture of origin
    a. Strongly agree
    b. Mostly agree
    c. Neutral
    d. Mostly disagree

e. Strongly disagree

15. My cultural values create strong ties between myself and my family
    a. Strongly agree
    b. Mostly agree
    c. Neutral
    d. Mostly disagree
    e. Strongly disagree

Sexual and Reproduction Health Questions

16. Are you currently sexually active?
    a. Yes
    b. No
17. I am sexually attracted to:
    a. Women
    b. Men
    c. Both
    d. Other
18. Have you ever had a sexually transmitted infection?
    a. Yes
        - ❑ Which?
        - ❑ What did you do about it?
        - ❑ How long did was it before you sought help?
            1. 1 – 3 days
            2. 4 – 7 days
            3. 8 – 14 days
            4. 15 – 30 days
            5. More than 30 days
        - ❑ Why did it take this long to get help?
            1. I didn't know I had an STI
            2. I was not sure what was wrong
            3. I was hoping the problem would pass
            4. I tried other methods to treat things first

5. Other (please specify) ______________________

b. No

19. Have you ever experienced an unplanned pregnancy? (This includes males who have impregnated someone and females who have been impregnated)

a. Yes

❑ How many times? _______ (If more than once the second question repeats accordingly)

❑ What happened with the pregnancy?

1. Had the baby
2. Terminated the pregnancy
3. Organised for adoption

b. No

20. Do you regularly use a form of contraception? (for those who are currently sexually active)

a. Yes

❑ Which? _________________________________

b. No

❑ Why not? (choose all that apply)

1. My partner and I are trying to conceive
2. It is against my cultural and/or religious beliefs to use them
3. I am not concerned about pregnancy at the moment
4. Other (please specify) ______________________

21. Do you regularly use condoms, dental dams or gloves to protect yourself, or others, from sexually transmitted infections?

a. Yes

❑ Which forms? ________________________

b. No

❑ Why not? (choose all that apply)

1. I am in a relationship and I trust my partner is safe
2. It is against my cultural and/or religious beliefs to use them
3. Other (please specify) ______________________

22. If you were having a sexual and reproductive health concern, how likely is it that you would seek help from the following people/places? Please indicate your response by clicking on the number that best describes your intention to seek help from each help source that is listed.

1 = Extremely Unlikely 3 = Unlikely 5 = Likely 7 = Extremely Likely

a. Intimate partner (e.g., girlfriend, boyfriend, husband, wife, de facto partner)	1	2	3	4	5	6	7
b. Friend (not related to you)	1	2	3	4	5	6	7
c. Parent	1	2	3	4	5	6	7
d. Other relative/family member	1	2	3	4	5	6	7
e. Sexual health clinic	1	2	3	4	5	6	7
f. The internet	1	2	3	4	5	6	7
g. Doctor/GP	1	2	3	4	5	6	7
h. Community, cultural or religious leader	1	2	3	4	5	6	7
i. I would not seek help from anyone	1	2	3	4	5	6	7
j. I would seek help from another not listed above (please list in the space provided, (e.g., work colleague. If no, leave blank)______________________	1	2	3	4	5	6	7

23. If you had a sexual and/or reproductive health concern what might stop you from seeking help from a services which specialise in sexual and reproductive health? (choose all that apply)
    a. I don't know where these services are
    b. The risk that my family or community could possibly find out that I had visited such a service
    c. These services do not cater well to people of my ethnic, cultural or religious group
    d. These services cost too much money
    e. These services are too far away from where I live
    f. Service trading hours
    g. I have other ways of getting support and assistance for such concerns
    h. Other (please specify) ______________________________
24. If you had a sexual and/or reproductive health concern what would facilitate seeking help from a services which specialise in sexual and reproductive health? (choose all that apply)
    a. Being made aware of where the services are
    b. Being confident that no one would find out
    c. Knowing that there are health workers who know how to cater services to people of my ethnic, cultural or religious group
    d. Services which are free or low cost
    e. Services which are close to where I live

f. Trading hours which include evenings and weekends

g. Other (please specify) ______________________

25. Do you have any other comments on anything related to the use of sexual and reproductive health services by migrants and their descendants?

____

____

____

____

NB: Online Q sorting activity then begins

NB: Online Q sorting activity ends and participants are asked if they would like to participate in phase two of the study:

Would you like to participate in a face-to-face interview to discuss your responses to the online questions and Q sort as well as share your perspective on 1.5 generation migrant sexual and reproductive health? Following participation in the interview you will receive $25 cash for your time.

- o Yes
  - o Please provide your full name ________________________
  - o Please provide your phone number ______________________
  - o Please provide your email address ______________________
- o No

Thank you for your helping with this important research ☺
